# Mental distress, perceived need, and barriers to receive professional mental health care among university students in Ethiopia

**DOI:** 10.1186/s12888-020-02602-3

**Published:** 2020-04-25

**Authors:** Assegid Negash, Matloob Ahmed Khan, Girmay Medhin, Dawit Wondimagegn, Mesfin Araya

**Affiliations:** Department of Psychiatry, College of Health Sciences, School of Medicine, Addis Ababa University, Addis Ababa, Ethiopia

**Keywords:** Mental distress, Perceived need, Barrier, Professional mental health care, Ethiopia, Undergraduate students

## Abstract

**Background:**

There is limited evidence on the extent of the perceived need and barriers to professional mental health service delivery to university students with mental distress in low- and middle-income countries (LMICs). This study was designed to assess the prevalence of mental distress, perceived need for professional mental health care and barriers to the delivery of services to affected undergraduate university students in Ethiopia.

**Methods:**

A multi-stage sampling technique was used to recruit 1135 undergraduate university students. Symptoms of mental distress were evaluated using the Self-Reported Questionnaire (SRQ-20) and a score of above seven was used to identify positive cases. The perceived need for professional mental health care was assessed using a single ‘yes or no’ response item and barriers to mental health care were assessed using Barriers to Access to Care Evaluation (BACE-30) tool. Percentage, frequency, mean, and standard deviation were employed to summarize demographic characteristics of the participants and to identify common barriers to mental health care service. Moreover, the association of demographic variables with total mean scores of BACE-III sub-scales was modeled using multiple linear regression.

**Results:**

The prevalence of mental distress symptoms was 34.6% and the perceived need for professional mental health care was 70.5% of those with mental distress. The top five barriers to receiving professional mental health service were (a) thinking the problem would get better with no intervention, (b) being unsure where to go to get professional help, (c) wanting to solve the problem without intervention, (d) denying a mental health problem existed, and (e) preferring to get alternative forms of mental care. Coming from a rural background, being a second and fourth-year student, and a family history of mental illness were significantly associated with barriers to receive professional mental health service.

**Conclusion:**

The high prevalence of mental distress, the paucity of mental health care, and the report of barriers to access what professional mental health care there is among Ethiopian undergraduate students is a call to address the disparity.

## Background

Mental distress is among the most common type of experience that accompanies mental health problem characterized by a mixture of different complaints such as feeling sad, worried, tense or angry [1]. Common mental disorders are a collective noun for anxiety, depression, and somatoform disorders that can adversely affect individuals across the world [2, 3]. According to the World Health Organization [4] 2015 report, over 300 million (4.4%) and 264 million (3.6%) of the total word population are estimated to suffer from anxiety and depression, respectively [5]. In particular, the contribution of these disorders to the global mental health burden from low- and middle-income countries (LMICs) is high [6]. However, the accessibility of mental health service is still very low, which accounts for the 76–85% treatment gap [7]. This gap has been linked to the lack of skilled human resources, lack of mental health policies, lack of access to mental health services, poverty, the preference for informal treatments, a lack of mental health literacy, the fear of stigma, and a low commitment from funders to access the services [8–12]. As a result, the majority of people living with anxiety and depression in LMICs do not receive professional mental health care [13].

As in any other LMICs, the prevalence and burden of anxiety and depression in Ethiopia is high. For example, a systematic review and meta-analysis study reported that the pooled prevalence of these disorders is 22% [14], which is associated with risk factors such as food insecurity [15], poverty, violence, migration, and substance use [16]. The burden of depression alone contributes to about 6.5% of the burden of diseases [16], however, as noted above few people are able to receive formal mental health services. Evidence showed that the pooled prevalence of help-seeking behaviors of people with depression is 38% [17]. In Ethiopia, most people with mental illness first contact non-professional care providers such as religious leaders and herbalists [18]. But, if the patient remains affected, he/she will go to western trained psychiatric care providers [18]. The psychiatric services are mainly concentrated in the capital city of Ethiopia, Addis Ababa [19].

To scale-up the limited mental health services across the country, the government of Ethiopia has planned to expand 100% of mental health care by 2020 [20]. The National Mental Health Strategy was developed in 2012 by the Federal Ministry of Health aimed to decentralize and integrate mental health services at the primary health care level [19]. At the university level, mental health services have been established to support students with mental health problems, although the quality of the service provided is under question. Currently, Ethiopia has 45 public universities, where 392,788 (255,657 male and 137,131female) undergraduate students enrolled in 2017. These students are adolescents, economically dependent on their family and full-time learner, they came from rural-urban backgrounds with diversified cultures, languages, and ethnicity.

The prevalence of mental distress is high among university students [21]. A cross-cultural web-based survey of 17,348 university students from 23 high-middle-and low-income countries reported that the average depression prevalence is 20% [22]. Another study also reported that the prevalence of depression and anxiety is 68.5 and 54.4%, respectively [23]. Similarly, the prevalence of these disorders ranges from 21.6–49% among Ethiopian university students [24, 25]. This high prevalence is associated with several factors including: (i) vulnerability of adolescence age for early onset of mental distress [26]; (ii) new identity formation [27]; (iii) challenges of being away from home for the first time [28]; and (iv) academic pressure, substance use [29], and financial difficulties [30]. Moreover, family histories of mental illness, conflicts with friends, not attending religious services, and being freshman are risk factors for mental distress among university students in Ethiopia [31, 32]. On the other hand, having high social support and enough pocket money are protective factors from mental distress [32].

Mental distress has a negative impact on university students’ academic performance [29]. Evidence shows that mentally distressed students scored poor examination result compared with non-distressed students [33]. Although mental distress has such impact, the treatment gap remains large ranging from 37 to 84% [34]. This treatment gap is also high among Ethiopian university students, where majority of the students receive treatment from informal sources such as family, friends, relatives, and religious leaders [35]. There are several barriers that hinder students with mental distress from receiving mental health services. Among these: (i) receiving help from friends or family; (ii) preferring to manage mental illness by self; (iii) normalizing mental illness; (iv) thinking that mental illness would get better by itself [36]; (v) lack of perceived need; (vi) being unaware of the existence of professional mental health services; (vii) fear of stigma, concerns about privacy; (viii) skepticism about treatment effectiveness; (iv) socio-economic problem [34]; and (x) denying mental illness [37].

There are associations between demographic variables and perceived need for mental health care. Female students have more positive attitudes to the utilization of mental health services compared with male students; the possible explanations could be that women experience more mental distress and they give more value for support received from professionals [38]. Conversely, male students are more likely to seek mental health care compared with female students; this might be caused by the interaction effect of other demographic variables in the analysis model [39]. Despite this, there is a finding reporting gender is not a predictor for seeking mental health care; possibly this is caused by insignificance gender difference in mean scores of depression and self-esteem [40]. Likewise, there is no gender difference in reporting barriers to receive mental health care [41]. Older students are more likely to have positive attitudes toward seeking mental health care than younger students; possibly caused by the past mental health care received [42]. Adolescents have a more positive attitude to seek mental health care than adults, because adolescents had more confidence in and better experience of using modern mental health care [39]. There are also insignificant difference in reporting barriers to access mental health care based on age [41].

Class years, family history of mental illness, and substance use are also reported as predictors to receive mental health care. For instance, first and fourth-years students are less likely to use mental health services compared with second and third-years students [43], although there is no change based on rural-urban backgrounds [44]. However, students who had personal contact with someone with a history of mental illness were significantly associated with decreased help-seeking intention; this could be possibly due to the negative experiences students had with a person who they know to have a mental illness [45]. Moreover, students with mental distress use substances to manage feeling of discomfort, which might hinder their interest or preparedness in seeking mental health care [46]. Mental distress, predictors, and barriers to receiving mental health care among university students occur globally, however, there might be higher prevalence, more complex stressors, lower help-seeking behaviors, and a higher treatment gap in LMICs compared with developed countries [47]. For example, even if the prevalence of mental distress is high among university students in LMICs, the majority of them do not receive professional mental health care [48, 49].

Most studies conducted in Ethiopian universities are primarily focused on assessing the prevalence of mental distress rather than looking at the possible barriers to receiving professional help. Besides of this, there is a literature gap with regard to identifying the perceived need for mental health service and demographic factors associated with barriers to receive mental health care among university students in Ethiopia. Therefore, this current study is aimed to assess the prevalence of mental distress, perceived need, and identify common barriers to receive professional mental health care among undergraduate students in Wolaita Sodo University (WSU). Our study also investigated the demographic predictors of the barriers to mental health care. The current study findings will fill the literature gap on professional help-seeking intention, predictors, and barriers to receive mental health service among undergraduate students in LMICs. Besides this, our findings inform to adapt and study feasibility of psychological intervention for students with mental distress within Ethiopian universities with potential implication for other LMICs universities.

## Methods

### Study setting

The current study is conducted at WSU, a public university located in the Sodo town of Wolaita Sodo Zone, Southern Nations, Nationalities, and Peoples’ Regional State (SNNPR) of Ethiopia. Sodo town is located 320 km south of Addis Ababa. WSU was established in 2007 as a result of the rapid expansion of higher education in Ethiopia. The university began with an intake of 801 students (609 males and 192 females) in four faculties and sixteen departments. Currently, the university runs undergraduate and graduate programs in six colleges and five schools. During the study period, a total of 12,028 (7321males and 4707 females) undergraduate students were registered. These students qualify to join the university by taking the Grade 12 national entrance examination prepared by Ministry of Education. WSU has two counseling offices and two health centers. There are three psychologists in the counseling offices that provide counseling services for students with mental health problems. The two health care centers are the Ottona hospital and the students’ clinic, both of which provide health care services. Ottona hospital is a referral hospital that provides health care services for the community and for the students with severe mental health problem by providing medication.

### Study design, objectives and study period

An institution-based cross-sectional survey was conducted among WSU undergraduate students from December 2017 to January 2018. The objective was to estimate the prevalence of mental health problems, perceived need, and to identify barriers and demographic predictors to receive professional mental health care.

### Sample size

A sample size of 1135 was estimated with an assumed prevalence rate of mental distress 40.9% [32], precision of ±3, 95% confidence interval, and 10% non-response considered. For the other two objectives (perceived need and barriers to receive mental health care) separate sample sizes were not estimated. All the participants who were screened positive for mental distress (> 7) were used as the denominator to estimate the proportion of students having a perceived need for professional mental health care. Those participants who had mental distress symptoms and who did not receive mental health services from professionals in the past 3 months were eligible to be part of the study.

### Sampling and procedures

A stratified multi-stage sampling technique was used to recruit study participants. A list of students’ names from all first to fifth years was obtained from the registrar office of WSU. The first participant’s name was selected randomly; the remaining participants were selected using systematic random sampling. To accomplish, the first step was stratifying undergraduate students by their schools/colleges (six colleges and five schools). For the second step, the total sample size was allocated into the 11 strata using probability proportional to the number of the students as a measure of size. The third step was selecting participants from each school and college based on the proportion of the size of each department. The fourth step was selecting participants from first to fifth-years based on the proportion to each year. Then, the final step was randomly selecting the first participant and systematically selecting the rest participants from each level and section of the study year.

### Measurements

The survey questionnaire consisted of four parts: Demographic Characteristic Questionnaire: used to document variables including participants’ sex, age, religion, ethnicity, marital status, current place of living, area where they grew up, level of the study years, family history of mental illness, and substance use.

Self-Reported Questionnaire (SRQ-20): It is a screening tool for mental distress developed by WHO [50]. SRQ-20 is a self-report instrument with 20 binary responses (yes/no) questions. It has the potential of detecting cases and non-cases with sensitivity ranging from 63 to 90 and specificity ranging from 44 to 95 [51]. WHO recommends SRQ-20 as a reliable and valid instrument to detect general Common Mental Disorders [51]. It was developed specifically for use in LMICs [50]. SRQ-20 has been previously translated into Amharic language in Ethiopia, locally validated [52, 53], and used in different community [54–56] and institution-based surveys [24, 25, 32, 57] with cut-off points ≥ 4 [57], ≥ 7 [25], ≥ 8 [32] and ≥ 11 [24]. SRQ-20 has good psychometric properties (i.e. sensitivity 86% and specificity 84%) for detecting individuals with mental distress in the Ethiopian population with an optimal cut-off point at ≥8 [58]. To identify cases in the current study, a cut-off point of > 7 was used based on a previous validation study of SRQ-20 in Ethiopia that resulted in good sensitivity and specificity using a cut-off point of 8 [58]. The pilot data collected from 38 undergraduate students in a similar population but in a different setting to the current study showed that the internal consistency of SRQ-20 was 0.77.

The Perceived Need for Professional Mental Health Care Questionnaire: Used to assess the perceived need for professional mental health services in the past 3 months. It has been used in the previous studies [59, 60]. The question is phrased as follows: ‘Was there a time when you thought you should see a doctor, counselor or other health professionals for your mental distress, but you did not go in the past three months?’ with the response options of Yes/No. “Yes” response implies the perceived need for mental health care but not received in the past 3 months, whereas “No” response implies no need for mental health care for mental distress. Therefore, the perceived need for professional mental health care in this study implies the number of students who reported “Yes” option.

Barriers to Access to Care Evaluation (BACE-III): BACE was originally developed to identify barriers to receive professional mental health service for people with mental health problems [61]. It has 30 items to be completed by the participant (self-complete measure). This instrument has good psychometric properties (i.e. validity, reliability, and acceptability) [61]. BACE-III has three dimensions of potential barriers of stigma (12 items), attitudinal (10 items) and instrumental (8 items) related. This instrument asks about a range of issues that have ever stopped, delayed or discouraged an individual from receiving professional care for a mental health problem in the past 3 months. The response scale ranges from 0 (not at all) to 3 (a lot); the higher score indicating a greater barrier. Five of the thirty items contain a fifth option: “Not applicable”. Findings for each barrier are presented in three ways: mean score for the item, barrier to any degree (the percentage of answering 1, 2 or 3) or major barrier (the percentage of answering 3) based on BACE-III manual for researchers.

For the current study, BACE-III was translated into the Amharic language by two Amharic language experts whose first language is Amharic and their second language is English. One expert who knows the subject matter translated the instrument based on the BACE-III translation guide. The masked back-translation was made by two English language experts and one mental health expert. The research team compared the back-translated instrument with the original version of BACE-III and agreed upon the consistency of the translation. The translated BACE-III was piloted on 40 undergraduate students in a similar population but in a different area of the current study setting. Its internal consistency was 0.85.

After the pilot study, the authors examined the applicability of each question in the university set-up and noticed that item number 27 and 28 need some modifications. Discussion was made with a mental health expert who has experience of adapting mental health instruments. Then, question number 27 which says ‘difficulty taking time off work’ was modified as ‘difficulty taking time off education’ and question number 28 which says ‘concern about what people at work might think, say or do’, was modified as ‘concern about what students might think, say or do’. The final version of the instrument was administered to students who scored > 7 on the SRQ-20 and who had a need (those who answered “Yes”) to receive professional mental care in the past 3 months of the study period. The internal consistency of the overall BACE-III scale after the revision was 0.85, whereas for stigma sub-scale = 0.83; attitudinal sub-scale = 0.67 and instrumental sub-scale = 0.60. Those participants who answered “No” for the perceived need for mental health care measuring questionnaire were asked ‘In the past three months, did you receive help from a psychologist, doctors, friends, family, religious leaders or traditional healers?’ by skipping the BACE-III questionnaire. See supplementary file 1.

### Training of data collectors and data collection procedures

Classroom representatives served as data collectors. A half day training was given by the principal investigator to data collectors about the aim of the research, the contents of data collection tools, how to approach participants, ethical issues, and responsibility of controlling missing data. The classroom representatives both males and females were contacted by the researcher through the help of their department heads, because they had cell phone numbers of each classroom representative. Then, with the assistance of the classroom representatives, the student participants came to the selected lecture halls and classrooms and the data collectors explained the aim of the study. Finally, after verbal agreement was received, the data collectors started to collect the data by explaining the instructions of all questionnaires with the close supervision of the principal investigator. To protect the confidentiality of the participants, personal identifiers were not included in the questionnaires; instead, a code was applied.

The data collection was carried out before the students’ final examination to control for an inflation of the prevalence of mental distress. Those who scored > 7 on SRQ-20 were asked to answer the questions about the perceived need for professional mental health care and then answer questions in the BACE-III questionnaire. Participants who answered “No” the question about the perceived need for professional mental health care skipped the BACE-III and answered why they did not need mental health treatment in the past 3 months. Finally, after the participants completed the self-administered questionnaires, the data collectors immediately checked the existence of incomplete and missed information before the participants left the room.

### Data analysis

Data cleaning and cross-checking were done before analysis using Statistical Packages for the Social Sciences (SPSS version 20). Descriptive statistical measures (i.e. percentage, frequency, mean, and standard deviation) were employed to summarize demographic characteristics of the participants and to identify barriers to mental health care services. Pearson chi-square test was used to examine the association between demographic variables with mental health care seeking intention and with the five most commonly reported barriers to receive mental health services. Furthermore, multiple linear regression was also used to model the association between demographic variables with a mean score of BACE-III sub-scales. Univariate regression analysis was used to identify potential candidate variables for multivariable linear regression with a *p*-value of < 0.2 by referring previous published articles [62, 63]. Then, further analysis was carried out using multivariable linear regression. The result was reported as being statistically significant whenever the p-value is less than 0.05.

### Ethical considerations

Ethical clearance for the conduct of the study was obtained from the Institutional Review Board (IRB) of Addis Ababa University College of Health Sciences. Information sheet containing details of the research and rights of the participants was attached to the questionnaire. Oral informed consent was obtained from the participants after we explained to them the purpose of the study, the participation was voluntary, and personal identifiers were not included in the questionnaires. Finally, the obtained data were kept anonymous and confidential during all stages of the research.

## Results

### Demographic characteristics

A total of 980 undergraduate students completed the screening phase survey from the sample of 1135 students approached, yielding 86.34% response rate. One third (34.6%) of the participants had scored > 7 on SRQ-20. The majority (60.5%) were male. The age of the participants ranged from 17 to 38 years with a mean age of 21.53 years (SD = 2.42). The participants were from diverse ethnic groups, the majority were from Amhara (34.6%) and Wolaita (20.9%) ethnic groups. Regarding marital status, 82.8% were single and 95.3% were living in the campus. Over half (54.7%) were from urban backgrounds. First-year, second-year, and third-year undergraduate students took 27.7, 26.6, and 25.9% of the sample, respectively (Table 1).

**Table 1:** Demographic characteristics of the study sample

Variables	Total Sample (*n*) % N (980)	Screened positive for mental distress (n) % N (339)	Participants with mental distress who have not received formal Mental Care % N (239)
Sex			
Male	593 (60.5)	176 (51.9)	127 (53.1)
Female	387 (39.5)	163 (48.1)	112 (46.9)
Age			
Mean	21.53	21.21	21.22
SD	2.42	1.95	1.82
Minimum	17	18	18
Maximum	38	30	28
Religion			
Christian Orthodox	543 (55.4)	241 (71.1)	164 (68.6)
Christian Protestant	330 (33.7)	50 (17.7)	46 (19.2)
Islam	80 (8.2)	30 (8.8)	22 (9.2)
Christian Catholic	8 (0.8)	2 (0.6)	2 (0.8)
No religion	8 (0.8)	4 (1.2)	3 (1.3)
Others	11 (1.1)	2 (0.6)	2 (0.8)
Ethnicity			
Amhara	339 (34.6)	164 (48.4)	110 (46.0)
Oromo	155 (15.8)	58 (17.1)	41 (17.2)
Wolaita	205 (20.9)	44 (13)	35 (14.6)
Gurage	80 (8.2)	24 (7.1)	15 (6.3)
Tigre	24 (2.4)	11 (3.2)	8 (3.3)
Sidama	58 (5.9)	9 (2.7)	7 (2.9)
Hadiya	32 (3.3)	8 (2.4)	6 (2.5)
Gamogofa	30 (3.1)	8 (2.4)	6 (2.5)
Others	57 (5.7)	13 (3.9)	11 (4.6)
Marital status			
Single	811 (82.8)	268 (79.1)	194 (81.2)
In a relation	114 (11.6)	50 (14.7)	31 (13.0)
Married but not living together	35 (3.6)	11 (3.2)	9 (3.8)
Divorced	14 (1.4)	7 (2.1)	3 (1.3)
Married and living together	6 (0.6)	3 (0.9)	2 (0.2)
Residence			
In Campus	934 (95.3)	320 (94.4)	223 (93.3)
Off Campus	20 (2)	8 (2.4)	7 (2.9)
Both	26 (2.7)	11 (3.2)	9 (3.8)
Area of growing			
Urban	536 (54.7)	176 (51.9)	127 (53.1)
Rural	444 (45.3)	163 (48.1)	112 (46.9)
Level of study year			
First-year	271 (27.7)	117 (34.5)	81 (33.9)
Second-year	261 (26.6)	85 (25.1)	58 (24.3)
Third-year	254 (25.9)	84 (24.8	67 (28.0)
Fourth-year	96 (9.8)	28 (8.3)	18 (7.5)
Fifth-year	98 (10.0)	25 (7.4)	15 (6.3)
Family history of mental illness			
Yes	67 (6.8)	34 (10.0)	22 (9.2)
No	913 (93.2)	305 (90.0)	217 (90.8)
Substance use			
Yes	58 (5.9)	39 (11.5)	31 (13.0)
No	922 (94.1)	300 (88.5)	208 (87)

### Mental distress

**Fig. 1:**
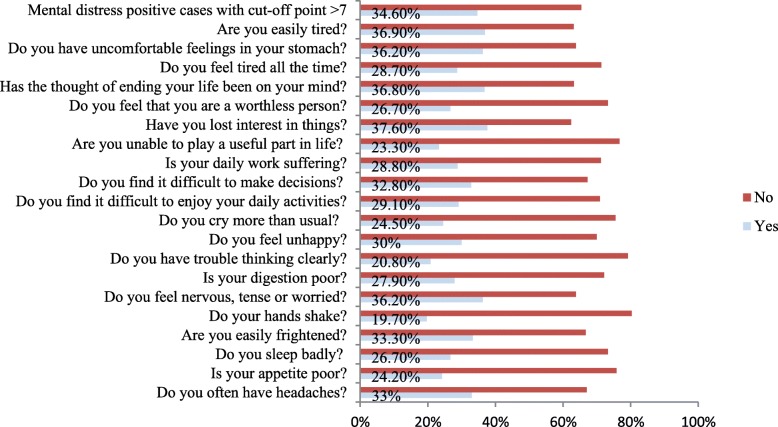
Prevalence of mental distress and its distribution of specific symptom

The prevalence of mental distress was 34.6%, indicated by 339 participants with SRQ-20 scored higher than 7. It was slightly higher (51.9%) among male students. The item-based response of the study participants to the SRQ-20 is summarized in Fig. 1. The top three frequently reported symptoms were: loss of interest in things (37.60%), being tired (36.90), and thought of ending one’s life (36.80%). The least reported symptom was handshaking/hand trembling (19.7%).

### Perceived need for professional mental health care

Of 339 participants with elevated symptoms of mental distress, 70.5% (*n* = 239) reported a perceived need for professional mental health care in the past 3 months. The remaining 29.5% did not report a need for professional mental health treatment, because they have received the service from informal sources (25.5% from religious leaders, friends, family, and traditional healers) and formal sources (4% from doctors and psychologists). There was no significant gender difference in seeking mental health service, χ2 (1) = 0.48, *p* = 0.49. Likewise, there were no significant differences among the remaining demographic variables in those seeking mental health services (Table 2).

**Table 2:** The association between demographic variables and perceived need for professional mental health care

Variables	Need for professional mental health care		χ 2	*P*-value
	Yes (*n*)	No (*n*)		
Sex				
Female	112	51	0.48	0.49
Male	127	49		
Religion				
Orthodox	164	77	3.02	0.39
Protestant	46	14		
Muslim	22	8		
Others	7	1		
Ethnicity				
Amhara	111	53	3.77	0.88
Oromo	41	17		
Wolaita	35	9		
Gurage	15	9		
Hadiya	6	2		
Tigre	8	3		
Sidama	7	2		
Gamogofa	6	2		
Others	10	3		
Marital status				
Single	193	75	6.83	0.15
In a relationship	31	19		
Married but no living together	2	1		
Divorced	10	1		
Married and living together	3	4		
Residence				
In campus	223	97	1.89	0.39
Off campus	7	1		
Both	9	2		
Area of growing				
Rural	112	51	0.48	0.49
Urban	127	49		
Level of study year				
First-year	81	36	5.61	0.23
Second-year	58	27		
Third-year	67	17		
Fourth-year	18	10		
Fifth-year	15	10		
Family history of mental illness				
No	217	88	0.61	0.44
Yes	22	12		
Substance use				
No	208	92	1.71	0.19
Yes	31	8		

### Barriers to receive professional mental health care for mental distress

Of the 339 participants who screened positive for mental distress, 239 (127 male and 112 female) had not received mental health services in the past 3 months, because of the barriers to receive the treatment, although they desired mental health care as indicated in Table 3. This table shows mean scores of an individual item, standard deviation, percentage to any degree, and major barriers to receive mental health care. There were top five barriers to receiving mental health care the reported percentage was greater than 60% to any degree (sum of responses of a little, quite a lot, and a lot).

**Table 3:** Barriers to receiving professional mental health care among students with mental distress who have not received mental care in the past three months (*n* = 239)

Barriers to Mental Health Care	Mental Distress who did not receive professional mental health treatment (N = 239)		Total (N)	Item Mean and (SD)
	Barrier to any degree % (*n*)	Major barrier % (*n*)		
Stigma-related barriers				
Concern about what my family might think, say, do or feel	48.1 (115)	18.4 (44)	239	0.98 (1.18)
Concern that I might be seen as weak for having a mental health problem	38.9 (93)	14.2 (34)	239	0.76 (1.10)
Feeling embarrassed or ashamed	29.8 (71)	10.9 (26)	239	0.58 (1.01)
Concern that I might be seen as ‘crazy’	31.9 (76)	10.5 (25)	239	0.61 (1.02)
Not wanting a mental health problem to be on my medical records	26.0 (62)	8.4 (20)	239	0.50 (0.95)
Concern that people might not take me seriously if they found out I was having professional care	28.0 (67)	7.1 (17)	239	0.51 (0.93)
Concern that people I know might find out	28.4 (68)	6.7 (16)	239	0.47 (0.87)
Concern about what my friends might think, say or do	33.5 (80)	6.7 (16)	239	0.56 (0.92)
Concern about what students might think, say or do	31.4 (75)	6.3 (15)	239	0.53 (0.90)
Attitudinal-related barriers				
**Thinking I did not have a problem**	**67.4 (161)**	**38.1 (91)**	**239**	**1.59 (1.29)**
**Thinking the problem would get better by itself**	**74.4 (178)**	**36.8 (88)**	**239**	**1.65 (1.22)**
**Preferring to get alternative forms of care**	**66.5 (159)**	**34.3 (82)**	**239**	**1.51 (1.27)**
**Wanting to solve the problem on my own**	**71.1 (170)**	**28.0 (67)**	**239**	**1.50 (1.18)**
Preferring to get help from family or friends	58.6 (140)	22.2 (53)	239	1.20 (1.20)
Dislike of talking about my feelings, emotions or thoughts	38.0 (91)	9.6 (23)	239	0.69 (1.02)
Concerns about the treatments available (e.g. medication side effects)	36.8 (88)	9.6 (23)	239	0.65 (1.01)
Thinking that professional care probably would not help	30.9 (74)	7.5 (18)	239	0.54 (0.93)
Fear of being put in hospital against my will	21.4 (51)	7.1 (17)	239	0.41 (0.89)
Having had previous bad experiences with professional care for mental health	16.7 (40)	4.6 (11)	239	0.30 (0.76)
Instrumental-related barriers				
Not being able to afford the financial costs involved	56.0 (134)	25.5 (61)	239	1.23 (1.25)
Having no one who could help me get professional care	59.8 (143)	24.7 (59)	239	1.26 (1.22)
**Being unsure where to go to get professional care**	**71.6 (171)**	**21.0 (51)**	**239**	**1.36 (1.11)**
Difficulty taking time off education	55.2 (132)	17.6 (42)	239	1.10 (1.16)
Problems with transport or travelling to appointments	44.4 (106)	17.6 (42)	239	0.92 (1.18)
Being too unwell to ask for help	51.9 (124)	14.6 (35)	239	0.96 (1.11)
Professionals from my own ethnic or cultural group not being available	26.4 (63)	6.7 (16)	239	0.47 (0.89)

*Note*: Question number 5, 14, 24, and 29 in the BACE-III were not included in this table, because more than 97% of the participants responded “Not applicable” option for each item. Barriers reported percentage greater than 60% to any degree were indicated in bold color

The first barrier to seeking mental health care was ‘thinking the problem would get better by itself’ reported by 74.4% to any degree and 37% thought that it would act as a major barrier to receive mental health services. The second was ‘being unsure where to go to get professional care’ which accounted for 71.6% to any degree and 21% reported as a major barrier. The third was ‘wanting to solve the problem on their own’, whereby 71% of the participants reported this as a barrier to any degree and 28% thought that it would act as a major barrier to receiving mental health care. The fourth was ‘denying a mental health problem’, where 67.4% of the participants reported this as a barrier to any degree and 38% reported it as a major barrier. The fifth was ‘preferring to get alternative forms of care’ reported as any degree of the barrier by 67%, while 34% of the participants reported it as a major barrier to receiving mental health service. Of all the demographic variables, only a family history of mental illness had a significant association, X^2^ [3] = 14.48 = *p* = 0.01 with ‘denying mental health problem’ of the top five barriers. See supplementary file 2.

Of the top five barriers, the top four were attitudinal related barriers to receiving professional mental health services. The fifth, ‘being unsure of where to get professional care’ was an instrumental-related sub-scale of BACE-III. As a result of, the mean score of attitudinal related barriers sub-scale of BACE-III (M = 1.26, SD = 0.68) was the highest when compared with instrumental related barriers sub-scale (M = 0.78, SD = 0.43) and stigma related barriers sub-scale (M = 0.61, SD = 0.65) of BACE-III.

### Predictors of attitudinal related barriers to receive professional mental health care

In univariate regression analysis (Table 4), fourth-year students perceived significantly more attitudinal related barriers (β = 0.27; 95%CI = 0.24, 1.16; *p* = 0.003) than the fifth-year students. Multivariable analysis also showed that only fourth-year students perceived significantly more attitudinal related barriers (β = 0.27; 95% CI = 0.21, 1.14; *p* = 0.01) than the fifth-year students.

**Table 4:** Predictors of attitudinal related barriers to receiving professional mental health care in univariate and multivariable linear regression (*n* = 239)

Variables	Attitudinal related barriers					
	Univariate			Multivariable		
	Beta	95% CI	*P*-value	Beta	95% CI	*P*-value
Age	.09	−.02, .08	.18	.01	−.05, .06	.91
Level of study years						
First year	.01	−.36, .38	.97	01	−.41, .42	.97
Second year	.03	−.33, .43	.80	.03	−.35, .46	.81
Third Year	.11	−.20, .54	.37	.12	−.21, .57	.37
Fourth Year	.27	.24, 1.16	.003	.26	.21, 1.14	.004
Fifth Year (Ref.)	–	.80, 1.48	< 0.01			
Family mental illness history						
No (Ref.)	–	1.14, 1.32	< 0.01			
Yes	.12	−.01, .59	.06	.11	−.04, .56	.09
R2				0.08		

*Note*. Reference category results for multivariable were: β = 1.05; 95% CI: −.29, 2.40; *P* = 0.12. Ref. = Reference category for univariate regression analysis and *CI* Confidence Interval for β

### Predictors of instrumental related barriers to receive professional mental health care

In univariate regression analysis (Table 5), female students perceived significantly fewer instrumental related barriers (β = −.15; 95%CI = −.24, −.02; *p* = 0.02) than male students. Students from rural background perceived significantly more instrumental related barriers (β = 0.18; 95%CI = 0.04, 0.26; *p* = 0.01) than students from urban background. Fourth-year students perceived significantly more instrumental related barriers (β = 0.28; 95%CI = 0.17, 0.76; *p* = 0.002) than the fifth-year students. Students who reported a family history of mental illness perceived significantly more instrumental related barriers (β = 0.16; 95%CI = 0.05, 0.43; p = 0.01) than students who reported no family history of mental illness. Students who reported substance use perceived significantly more instrumental related barriers (β = 0.15; 95%CI = 0.03, 0.36; *p* = 0.02) than students who reported no substance use. A 1 year increase in age was associated with 0.19 unit increased in instrumental related barriers to receiving mental health services (β = 0.19; 95%CI = 0.02, 0.07; *p* = 0.004). In multivariable analysis, students from rural background perceived significantly more instrumental related barriers (β = 0.16; 95%CI = 0.03, 0.25; *p* = 0.01) than students from urban background. Besides this, second-year (β = 0.27; 95%CI *=* 0.02, 0.52; *p* = 0.03) and fourth-year students (β = 0.29; 95%CI *=* 0.19, 0.77; *p* = 0.001) perceived significantly more instrumental related barriers than the fifth-year students.

**Table 5:** Predictors of instrumental related barriers to receiving professional mental health care in univariate and multivariable linear regression (*n* = 239)

Variables	Instrumental related barriers					
	Univariate			Multivariable		
	Beta	95% CI	P-value	Beta	95% CI	*P*-value
Sex						
Male (Ref.)	–	.77, .92	< 0.01			
Female	−.15	−.24, −.02	.02	−.10	−.20, .02	.12
Age	.19	.02, .07	.004	.13	−.01, .07	.09
Area of growing						
Urban (Ref.)		.64, .78	< 0.01		.	
Rural	.18	.04, .26	.01	.16	.03, .25	.01
Level of study years						
First year	.11	−.13, .34	.39	.23	−.05, .47	.11
Second year	.14	−.10, .39	.25	.27	.02, .52	.03
Third Year	.14	−.10, .37	.27	.23	−.02, .47	.07
Fourth Year	.28	.17, .76	.002	.29	.19, .77	.001
Fifth Year (Ref.)		.42, .86	< 0.01			
Family mental illness history						
No (Ref.)		.70, .82	< 0.01			
Yes	.16	.05, .43	.01	.09	−.05, .32	.15
Substance use						
No (Ref.)		.70, .82	< 0.01			
Yes	.15	.03, .36	.02	.09	−.05, .28	.17
R2				0.14		

Note. Reference category for multivariable: β = −.16; 95% CI = −1.02, 0.70 *p* = .72. Ref. refers to reference category for univariate regression analysis and *CI* Confidence Interval for β

### Predictors of stigma related barriers to receive professional mental health care

Univariate regression analysis showed that students from rural background perceived significantly more stigma related barriers (β = 0.13; 95%CI = 0.00, 0.33; *p* = 0.05) than students from urban background. Students who reported a history of mental illness in the family perceived significantly more stigma related barriers (β = 0.13; 95%CI = 0.01, 0.57; p = 0.05) than students who reported no family history of mental illness. Students who reported substance use perceived significantly more stigma related barriers (β = 0.13; 95%CI = .00, 0.49; p = 0.05) than students who reported no substance use. A 1 year increase in age was associated 0.17 unit increased in stigma-related barriers to mental health services (β = 0.17; 95%CI = 0.02, 0.10; *p* = 0.01). In multivariable analysis, only fourth-year students perceived significantly more stigma related barriers (β = 0.24; 95%CI = 0.14, 1.01; p = 0.01) than the fifth-year students (Table 6).

**Table 6:** Predictors of stigma related barriers to receiving professional mental health care in univariate and multivariable linear regression (*n* = 239)

Variables	Stigma related barriers					
	Univariate			Multivariable		
	Beta	95% CI	P-value	Beta	95% CI	*P*-value
Age	.17	.01, .10	.01	.10	−.02, .09	.19
Area of growing						
Urban (Ref.)		.42, .65	< 0.01			
Rural	.13	.00, .33	.05	.12	−.02, .32	.08
Level of study years						
First year	.02	−.32, .37	.89	.11	−.25, .54	.47
Second year	−.002	−.36, .36	.99	.08	−.26, .50	.54
Third Year	.13	−.18, .54	.32	.18	−.11, .63	.16
Fourth Year	.24	.14, 1.01	.01	.24	.14, 1.02	.01
Fifth Year (Ref.)	–	.19, .83	< 0.01			
Family mental illness history						
No (Ref.)	–	.50, .67	< 0.01			
Yes	.13	.01, .57	.05	.09	−.09, −.48	.19
Substance use						
No (Ref.)		.49, .67	< 0.01			
Yes	.13	.00, .49	.05	.07	−.12, .39	.31
R2				0.10		

Note. Reference category for multivariable: β: −.45; 95% CI = −1.72 to .83; p = 0.49. Ref. refers to reference category for univariate regression analysis and *CI* Confidence Interval for β

## Discussion

In this study, there is high prevalence of mental distress and perceived need for professional mental health care services among university students. The top five frequently reported barriers to receive professional mental health service were: thinking the problem would get better by itself, being unsure where to go to get professional care, wanting to solve the problem by oneself, denying a mental health problem, and preferring to get alternative forms of care. Having a rural background, being a second and fourth-year student, and a family history of mental illness were significantly associated with barriers to receive professional mental health service.

The prevalence of mental distress which is reported in the present study is higher than what has been reported in the meta-analysis of the general population studies in Ethiopia [14]. Perhaps our finding is not surprising, because university students are more likely than the general population to be exposed to mental stress [21]. The possible difference between individual studies reviewed in the meta-analysis [14] and the present study could be partly attributed to the discrepancy in data collection instrument with cut-off points used, age group, and setting. The data collection tools used in the most individual article within the meta-analysis study were ICD-10, PHQ-9, EPDS, K10, HADS etc. with different cut-off points, but in our study we have used SRQ-20 that might be one cause for the discrepancy. The other was a difference in age group and setting, where young person experience higher mental distress compared with adults in the general population.

The current prevalence of mental distress is higher than that reported in previous studies conducted among university students [24, 64]. One possible reason for the discrepancy is the difference in the cut-off values used to define mental illness [24, 64]. The other explanation for the difference is other studies did not use locally validated instrument [64]. On the other hand, the present finding is lower than what was reported in previous studies in Ethiopian universities [35, 65]. The first possible justification for the difference might be data collection tool being used to screen mental distress [35, 65]. The second possible reason for the difference could be the timing of the data collection, where our data collected prior to the approaching final examinations. The present finding is comparable with a study report conducted in Jima University [66]. This might be resulted from similarity of the data collection tool and the cut-off points used to define mental distress.

The high prevalence of perceived need for professional mental health services in the current study suggests that most students with mental distress in Wolaita Sodo University remain untreated. This may not be surprising, because most universities in LMICs are ill-equipped to provide services for students’ mental health issues [47]. Previous study also reported only a few university students receive mental health services for their mental health problems as a result of lack of appropriate services [67]. Our finding is higher than previously reported in the general population of Ethiopia. For instance, a meta-analysis study reported that the pooled prevalence of the help-seeking intention of people with depression in Ethiopia is 42% [17], which is much lower than the current finding. The possible explanations for the difference could be a difference in mental health literacy, study population and the data collection instruments being used. Moreover, our study supports the previous web-based survey reporting that 37 to 84% of university students screened positive to mental distress did not receive any professional mental health service [34]. The similarity of the result may be due to using similar data collection tool and similar age group of participants.

Among the top five reported barriers to receive professional mental health service by the students who recognized a need for care, the first was thinking mental distress would get better by itself. This indicates that students perceive mental distress would get better without receiving any treatment, which may be associated with considering mental health problems as less serious so they are reluctant to use available mental health services [68] and it may also be associated with having poor mental health literacy [69]. The current finding supports a prior study reporting that the majority of college students believed that time by itself would solve their mental health problem [59].

Lack of information where to go to get professional care was reported as the second barrier to receiving mental health service in the University. However, WSU has two counseling offices and a teaching referral hospital that aim to provide mental health services for students with mental health problems. This information gap is probably caused by a lack of awareness creation of these services by the mental health service providers. Our finding is supported by prior studies, where the majority of university students had no information about the availability of mental health service in their university [70, 71].

Wanting to solve mental health problems by oneself is reported as the third common barrier to receive mental health service in the present study. This suggests that most students may not want to share their mental health problems with professionals preferring to handle the problem by themselves. This is possibly due to perceiving their problem as not serious or transitory, being skeptical about the effectiveness of professional mental health service, fear of stigma, and privacy issue [34]. As a result, they might prefer to manage their mental health problem alone perhaps utilizing both positive and negative strategies such as problem-solving [72], substance uses, and isolation [73] as examples. The present finding supports past studies reporting a major barrier to receiving formal mental health service among university students with mild to moderate depression and anxiety is preferring to self-medicate [74, 75].

The fourth barrier identified in the present study is denying mental health problems. Students may not want to recognize their mental health problems due to lack of knowledge about mental illness [69] or they may deny their mental health problem as a coping strategy by rejecting reality and not taking appropriate action to treat their problem [73]. Our finding support a prior study finding reporting that the majority of university students deny mental health problems which hindered them from receiving mental health care [37]. Furthermore, the present study interestingly showed that a family history of mental illness significantly associated with a student denying mental distress. This could be due to students had negative experiences by being with individual with mental illness previously so that they could deny their illness as a coping mechanism [45].

The fifth commonly reported barrier for using mental health service is preferring to get mental health service from informal sources. This suggests that majority of the students receive mental health help from friends, family, relatives, religious leaders, and traditional healers [35], which is also common practice in the general population of Ethiopia [76]. The present finding is also supported by previous studies, where informal sources of mental health care reported by college students was cited as a reason for not receiving mental health services at their university [69, 77].

Interestingly, the current study found that fourth-year students with mental distress are more likely than fifth-year students to report attitudinal, instrumental, and stigma related treatment barriers. Since, the majority of the fourth- and fifth-years students in the present study were from the engineering department, the possible difference could be resulted from as the level of study year/age increases, students become more stable and have better mental health literacy [78, 79]. In this sense, fourth-year students might encounter more barriers to receiving mental health care compared to fifth-year students. However, our finding contradicts a study finding first-year students are more likely than their third-year and senior students to perceive a greater number of barriers to receiving mental health care [37]. The result difference with the present study possibly due to the difference in the data collection tool, study setting, and sample size in each level of the study year. Caution in the present study, the number of the fourth-year students was small.

Our study also found that students from rural backgrounds are more likely than students from urban backgrounds to face instrumental related barriers to access professional mental health care. This might be because of young person from rural areas may not have a knowledge of mental illnesses so that they may not have sufficient information about the availability of free mental health services in the university and they may not be psychologically open toward professional mental health services [80]. Our finding compare positively with a study conducted in Australia reporting that adolescents from rural areas have more instrumental related challenges for receiving formal mental health care than adolescents from urban areas [81]. Our study also shows that second-year students reported more instrumental-related barriers compared with fifth-year students. This was probably due to the interaction effects of other controlled variables in the adjusted model. This may need further study in the future.

The present study implies that mental distress is prevalent among undergraduate students. Likewise, the need for mental health services is increasing, even though the students are not be able to receive the service provided in the university. This was because of attitudinal, instrumental and stigma-related barriers. Particularly, fourth-year students and students from rural background were more likely to report these barriers compared with fifth-year and students from urban backgrounds, respectively. This indicates to a need for designing practical mental health interventions to treat students’ mental distress, to alleviate their psychological suffering and minimize the effect on their academic work, general and social functioning [29]. Therefore, the present findings provide useful information and directions for university mental health service providers to create awareness about mental health problems and their service, the benefits of receiving mental health care, and when and where to seek mental health services by distributing flyers, preparing training, and mental health day. All these together enhance to develop active university-based mental health intervention to reduce the prevalence of mental distress and to satisfy the need for receiving mental health service by minimizing the reported major barriers.

Any research has its own limitations; similarly, the present study is not limitation free. First, data were collected using self-reported questionnaires so that recalling bias may occur for mental distress symptoms that happened in the past 1 month and rating the degree of barriers to mental health care may also difficult to remember. Second, the barriers to the provision of mental health care measuring instrument was not locally adapted, although it was properly translated and piloted for the present research. Third, a screening tool was used to identify participants positive for mental distress; it would have been better to use a diagnosis tool. Fourth, data collectors were classroom representatives, so that they were in a position to know the participants’ response to each item while checking missing on the questionnaires. Fifth, since the participants were recruited from a single public university, it is difficult to generalize the result to all public universities and private colleges that are found in Ethiopia. Finally, students who received treatment from the informal sources should have to complete BACE-III to understand whether they were aware of their needs for professional care or not, but we did not do that.

Further, future research is needed to study barriers to receiving professional mental health care among students with mental distress who do not wish to receive mental health care from professionals as a result of receiving treatment from informal sources. Additionally, the present study has also investigated some demographic predictors of barriers to receive mental health care, but further study is necessary to examine the associations of other variables such as mental health literacy and academic results with barriers to receive mental health services. Despite the limitations mentioned above, the present study has some strengths. First, a large number of students participated in the prevalence study. Second, the study used a locally adapted instrument, SRQ-20. Third, the research contains findings of the prevalence of mental distress, perceived needs, predictors, and barriers to receive professional mental health services together; all this information taken together can serve as input for future feasibility studies of mental health interventions for mental distress among university students.

## Conclusions

There is high prevalence of mental distress and perceived need for professional mental health service among undergraduate students at Wolaita Sodo University that identify the need for professional mental health interventions. Mental health providers in the university should make their services accessible to the students and promote the service for better utilization. Besides the interventions, developing preventive mental health education strategies is essential to address the prevalence of mental distress with the creation of conducive environments that promote and sustain positive mental health for every student. Moreover, preparing for the celebration of mental health day in the university can play a role in changing the attitude of students toward mental health care and improving mental health literacy, because out of the five major barriers, four of them were attitudinal related. In this celebration day, creating awareness about the treatment of mental distress like any other physical illness can be emphasized, as well as the benefits of receiving mental health care from professionals, recognizing mental distress in the early stages, and educating students to seek mental health care from professionals in parallel with receiving treatment from alternative sources. Therefore, this paper is a call for action from university administrations, university mental health care providers, and the Ministry of Science and Higher Education for helping undergraduate students with mental distress and to work to minimizing mental health distress on campus.

## Supplementary information

**Additional file 1.** Instruments used for data collection.

**Additional file 2.** The association between demographic variables and common barriers to receive professional mental health care.

## Data Availability

The datasets used and/or analyzed during the current study are available from the corresponding author on reasonable request.

## Abbreviations

BACE: Barriers to Access to Care Evaluation
LMICs: Low- and Middle- Income Countries
SNNPR: Southern Nations, Nationalities, and Peoples’ Region
SRQ: Self-Reported Questionnaire
SPSS: Statistical Packages for Social Sciences
M: Mean
SD: Standard Deviation
WHO: World Health Organization
WSU: Wolaita Sodo University
ICD-10: International Classification of Diseases 10th edition
PHQ-9: Patient Health Questionnaire
EPDS: Edinburgh Postnatal Depression Scale
K10: Kessler Psychological/Mental Distress Scale
HADS: Hospital Anxiety and Depression Scale
