# Supplementary material for: Mental distress, perceived need, and barriers to receive professional mental health care among university students in Ethiopia
Source: BMC Psychiatry. 2020 Apr 25;20:187. doi: 10.1186/s12888-020-02602-3 (PMC7183586; doi:10.1186/s12888-020-02602-3)
Supplement: Supplementary file 1 — Additional file 1. Instruments used for data collection. [file 12888_2020_2602_MOESM1_ESM.docx]

Addis Ababa University

College of Health Sciences, Department of Psychiatry

Title: Mental distress, perceived need, and barriers to receive professional mental health care among Wolaita Sodo University Students

Dear participants of this research, the main objective of the study is to assess the prevalence of mental distress, perceived need and barriers to receive professional mental health care. This questionnaire has four parts. Part I focused on assessing the demographic characteristics of the study participants. Part II aimed to assess the prevalence of mental distress. Part III is designed to assess the perceived need for professional mental health care. Part IV is aimed to assess barriers to receive professional mental health care. Thus, your genuine responses to each items listed in all parts of the questionnaires will help to provide important information for the indented study. Your responses will be kept completely confidential, and will be used for the purpose of this research only. All the data from this study will be in description form only, with no personal names or other identifiers. Therefore, be honest to answer the following questions and don’t hesitate to ask anything that needs clarification from the data collectors. I would like to thank you for participating in this study.

Part I. Demographic Information

Instruction: Please after reading the following questions carefully, tick the answer to be ticked in the given box and write your answer on the space provided for the rest questions.

1. Gender

Male Female

2. Age _____________________________________

3. Religion

Orthodox Christian Protestant Christian

Muslim Catholic Christian

No religion Other (specify*)*___________

4. Ethnicity

Amhara Oromo Wolaita Gurage

Hadiya Tigre Sidama Other Specify________

5. Marital status

Single In a relation Married and living together

Married but not living together Divorced Widowed

6. Current place of living

In campus

Off campus

Both

7. Area of growing up

Rural

Urban

8. Level of study year

First-year Second-year Third-year

Fourth-year Fifth-year Six-year

9. Family history of mental illness

Yes No

10. Do you use psycho-active drug/s and alcohol currently?

Yes No

Part II. Self-Reporting Questionnaire, SRQ-20

Instruction: The following questions are related to certain pains and problems that may have bothered you in the last one month. If you think the question applies to you and you had the described problem in the last one month, answer Yes. On the other hand, if the question does not apply to you and you did not have the problem in the last one month, answer No. Please, tick your answer on the space provided.

| No | Question | Answer | |
| --- | --- | --- | --- |
|  |  | Yes (1) | No (0) |
| 1 | Do you often have headaches? |  |  |
| 2 | Is your appetite poor? |  |  |
| 3 | Do you sleep badly? |  |  |
| 4 | Are you easily frightened? |  |  |
| 5 | Do your hands shake? |  |  |
| 6 | Do you feel nervous, tense or worried? |  |  |
| 7 | Is your digestion poor? |  |  |
| 8 | Do you have trouble thinking clearly? |  |  |
| 9 | Do you feel unhappy? |  |  |
| 10 | Do you cry more than usual? |  |  |
| 11 | Do you find it difficult to enjoy your daily activities? |  |  |
| 12 | Do you find it difficult to make decisions? |  |  |
| 13 | Is your daily work suffering? |  |  |
| 14 | Are you unable to play a useful part in life? |  |  |
| 15 | Have you lost interest in things? |  |  |
| 16 | Do you feel that you are a worthless person? |  |  |
| 17 | Has the thought of ending your life been on your mind? |  |  |
| 18 | Do you feel tired all the time? |  |  |
| 19 | Do you have uncomfortable feelings in your stomach? |  |  |
| 20 | Are you easily tired? |  |  |

Part III: Perceived need for professional mental health care

Instruction: Please tick your answer for the following questions.

1. Was there a time when you thought you should see a doctor, counselor or other health professionals or seek any other help for your mental distress, but you didn’t go in the past three months? Yes No

1.1. If your answer is No for question one, from whom did you receive mental health treatment?

Doctors University Counselors

Traditional healers Family

Friends Relatives

Religious leaders Didn’t receive

Student clinic nurses Other (specify)____________

Part IV: Barriers to Access to Care Evaluation

Below you can see a list of things which can stop, delay or discourage people from getting professional care for a mental health problem, or continuing to get help. By professional care we mean care from doctors, psychiatrists, counselors/psychologists or mental health nurses.

| Have any of these issues ever stopped, delayed or discouraged you from getting, or continuing with, professional care for a mental health problem? Please tick your answer | Yes |  |
| --- | --- | --- |
|  | No |  |

Please circle one number on each row to indicate the answer that best suits you. For ‘not applicable’ e.g. if it is a question about children and you do not have children, please cross the Not applicable box.

|  | Issue | This has stopped, delayed or discouraged me  NOT AT ALL | This has stopped, delayed or discouraged me  A LITTLE | This has stopped, delayed or discouraged me  QUITE A LOT | This has stopped, delayed or discouraged me  A LOT |
| --- | --- | --- | --- | --- | --- |
| 1. | Being unsure where to go to get professional care | 0 | 1 | 2 | 3 |
| 2. | Wanting to solve the problem on my own | 0 | 1 | 2 | 3 |
| 3. | Concern that I might be seen as weak for having a mental health problem | 0 | 1 | 2 | 3 |
| 4. | Fear of being put in hospital against my will | 0 | 1 | 2 | 3 |
| 5. | Concern that it might harm my chances when applying for jobs  Not applicable □ | 0 | 1 | 2 | 3 |
| 6. | Problems with transport or travelling to appointments | 0 | 1 | 2 | 3 |
| 7. | Thinking the problem would get better by itself | 0 | 1 | 2 | 3 |
| 8. | Concern about what my family might think, say, do or feel | 0 | 1 | 2 | 3 |
| 9. | Feeling embarrassed or ashamed | 0 | 1 | 2 | 3 |
| 10. | Preferring to get alternative forms of care (e.g. traditional / religious healing or alternative / complementary therapies) | 0 | 1 | 2 | 3 |
| 11. | Not being able to afford the financial costs involved | 0 | 1 | 2 | 3 |
| 12. | Concern that I might be seen as ‘crazy’ | 0 | 1 | 2 | 3 |
| 13. | Thinking that professional care probably would not help | 0 | 1 | 2 | 3 |
| 14. | Concern that I might be seen as a bad parent. Not applicable □ | 0 | 1 | 2 | 3 |
| 15. | Professionals from my own ethnic or cultural group not being available | 0 | 1 | 2 | 3 |
| 16. | Being too unwell to ask for help | 0 | 1 | 2 | 3 |
| 17. | Concern that people I know might find out | 0 | 1 | 2 | 3 |
| 18. | Dislike of talking about my feelings, emotions or thoughts | 0 | 1 | 2 | 3 |
| 19. | Concern that people might not take me seriously if they found out I was having professional care | 0 | 1 | 2 | 3 |
| 20. | Concerns about the treatments available (e.g. medication side effects) | 0 | 1 | 2 | 3 |
| 21 | Not wanting a mental health problem to be on my medical records | 0 | 1 | 2 | 3 |
| 22. | Having had previous bad experiences with professional care for mental health | 0 | 1 | 2 | 3 |
| 23. | Preferring to get help from family or friends | 0 | 1 | 2 | 3 |
| 24. | Concern that my children may be taken into care or that I may lose access or custody without my agreement  Not applicable □ | 0 | 1 | 2 | 3 |
| 25. | Thinking I did not have a problem | 0 | 1 | 2 | 3 |
| 26. | Concern about what my friends might think, say or do | 0 | 1 | 2 | 3 |
| 27. | Difficulty taking time off education | 0 | 1 | 2 | 3 |
| 28. | Concern about what students might think, say or do  Not applicable □ | 0 | 1 | 2 | 3 |
| 29. | Having problems with childcare while I receive professional care  Not applicable □ | 0 | 1 | 2 | 3 |
| 30. | Having no one who could help me get professional care | 0 | 1 | 2 | 3 |

Thank You So Much
