# Supplementary material for: Mental distress, perceived need, and barriers to receive professional mental health care among university students in Ethiopia
Source: BMC Psychiatry. 2020 Apr 25;20:187. doi: 10.1186/s12888-020-02602-3 (PMC7183586; doi:10.1186/s12888-020-02602-3)
Supplement: Supplementary file 2 — Additional file 2. The association between demographic variables and common barriers to receive professional mental health care. [file 12888_2020_2602_MOESM2_ESM.docx]

| **Variables** | **Common barriers to receive mental health care** | | | | | | | | | | | | | | | | | | | | | | | | | | | |  | |
| --- | --- | --- | --- | --- | --- | --- | --- | --- | --- | --- | --- | --- | --- | --- | --- | --- | --- | --- | --- | --- | --- | --- | --- | --- | --- | --- | --- | --- | --- | --- |
|  | **Thinking the problem**  **would get better by itself** | | | | **χ 2** | **P** | **Being unsure where to go**  **to get professional care** | | | | **χ 2** | **P** | **Wanting to solve the problem by own** | | | | **χ 2** | **P** | **Denying mental health problem** | | | | **χ 2** | **P** | **Preferring to get alternative forms of mental care** | | | | **χ 2** | **P** |
|  | **Not at all** | **A little** | **Quite a lot** | **A lot** |  |  | **Not at all** | **A little** | **Quite a lot** | **A lot** |  |  | **Not at all** | **A little** | **Quite a lot** | **A lot** |  |  | **Not at all** | **A little** | **Quite a lot** | **A lot** |  |  | **Not at all** |  | **Quite a lot** | **A lot** |  |  |
|  |  |  |  |  |  |  |  |  |  |  |  |  |  |  |  |  |  |  |  |  |  |  |  |  |  | **A little** |  |  |  |  |
| **Sex** |  |  |  |  | **3.2** | **0.37** |  |  |  |  | **1.22** | **0.75** |  |  |  |  | **4.34** | **.23** |  |  |  |  | **5.1** | **.2** |  |  |  |  | **3.70** | **.30** |
| Female | **34** | **21** | **16** | **41** |  |  | **33** | **34** | **21** | **24** |  |  | **32** | **27** | **28** | **25** |  |  | **41** | **17** | **12** | **42** |  |  | **36** | **14** | **19** | **43** |  |  |
| Male | **27** | **29** | **24** | **47** |  |  | **35** | **34** | **31** | **27** |  |  | **37** | **22** | **26** | **42** |  |  | **37** | **15** | **26** | **49** |  |  | **44** | **26** | **18** | **39** |  |  |
| **Religion** |  |  |  |  | **16.3** | **0.36** |  |  |  |  | **14.38** | **0.50** |  |  |  |  | **18.99** | **.21** |  |  |  |  | **18.3** | **.3** |  |  |  |  | **9.45** | **.85** |
| Orthodox | **41** | **30** | **31** | **62** |  |  | **41** | **44** | **40** | **39** |  |  | **48** | **35** | **39** | **42** |  |  | **53** | **20** | **27** | **64** |  |  | **54** | **25** | **26** | **59** |  |  |
| Protestant | **14** | **10** | **7** | **15** |  |  | **19** | **12** | **7** | **8** |  |  | **9** | **8** | **10** | **19** |  |  | **16** | **6** | **5** | **19** |  |  | **17** | **10** | **7** | **12** |  |  |
| Muslim | **6** | **5** | **2** | **9** |  |  | **6** | **10** | **3** | **3** |  |  | **10** | **5** | **1** | **6** |  |  | **6** | **5** | **3** | **8** |  |  | **6** | **3** | **3** | **10** |  |  |
| Others | **0** | **5** | **0** | **2** |  |  | **2** | **2** | **2** | **1** |  |  | **2** | **1** | **4** | **7** |  |  | **3** | **1** | **3** | **0** |  |  | **3** | **2** | **1** | **1** |  |  |
| **Ethnicity** |  |  |  |  | **15.95** | **0.77** |  |  |  |  | **26.61** | **0.18** |  |  |  |  | **19.24** | **.57** |  |  |  |  | **39.6** | **.0** |  |  |  |  | **24.68** |  |
| Amhara | **28** | **23** | **19** | **40** |  |  | **30** | **35** | **25** | **20** |  |  | **36** | **25** | **21** | **28** |  |  | **45** | **12** | **16** | **37** |  |  | **37** | **18** | **18** | **37** |  |  |
| Oromo | **8** | **8** | **6** | **19** |  |  | **9** | **11** | **11** | **10** |  |  | **9** | **8** | **11** | **13** |  |  | **9** | **4** | **9** | **19** |  |  | **10** | **6** | **7** | **18** |  |  |
| Wolaita | **10** | **7** | **8** | **10** |  |  | **11** | **9** | **8** | **7** |  |  | **9** | **4** | **8** | **14** |  |  | **9** | **6** | **4** | **16** |  |  | **14** | **7** | **7** | **7** |  |  |
| Gurage | **6** | **3** | **0** | **6** |  |  | **4** | **8** | **1** | **2** |  |  | **3** | **4** | **2** | **6** |  |  | **2** | **7** | **1** | **5** |  |  | **4** | **6** | **1** | **4** |  |  |
| Hadiya | **0** | **2** | **2** | **2** |  |  | **1** | **1** | **0** | **4** |  |  | **2** | **1** | **2** | **1** |  |  | **1** | **0** | **2** | **3** |  |  | **2** | **1** | **0** | **3** |  |  |
| Tigre | **3** | **5** | **2** | **4** |  |  | **8** | **1** | **1** | **4** |  |  | **3** | **6** | **4** | **1** |  |  | **8** | **0** | **4** | **2** |  |  | **7** | **2** | **3** | **2** |  |  |
| Sidama | **1** | **1** | **2** | **3** |  |  | **2** | **2** | **2** | **1** |  |  | **3** | **0** | **2** | **2** |  |  | **1** | **2** | **0** | **4** |  |  | **3** | **0** | **0** | **4** |  |  |
| Others | **5** | **1** | **1** | **4** |  |  | **3** | **1** | **4** | **3** |  |  | **4** | **1** | **4** | **2** |  |  | **3** | **1** | **2** | **5** |  |  | **3** | **0** | **1** | **7** |  |  |
| **Marital status** |  |  |  |  | **12.98** | **0.37** |  |  |  |  | **10.22** | **0.60** |  |  |  |  | **10.93** | **.54** |  |  |  |  | **10.84** | **.5** |  |  |  |  | **16.77** | **.16** |
| Single | **50** | **38** | **31** | **75** |  |  | **52** | **54** | **45** | **43** |  |  | **60** | **35** | **42** | **57** |  |  | **61** | **24** | **33** | **76** |  |  | **70** | **27** | **27** | **70** |  |  |
| In a relationship | **5** | **11** | **8** | **7** |  |  | **11** | **7** | **7** | **6** |  |  | **5** | **11** | **8** | **7** |  |  | **9** | **7** | **5** | **10** |  |  | **7** | **9** | **6** | **9** |  |  |
| Married but not living together | **4** | **1** | **1** | **3** |  |  | **2** | **5** | **0** | **2** |  |  | **3** | **2** | **3** | **1** |  |  | **4** | **1** | **0** | **4** |  |  | **3** | **2** | **3** | **1** |  |  |
| Divorced | **1** | **0** | **0** | **2** |  |  | **2** | **1** | **0** | **0** |  |  | **0** | **1** | **1** | **1** |  |  | **2** | **0** | **0** | **1** |  |  | **0** | **1** | **0** | **2** |  |  |
| Married and living together | **1** | **0** | **0** | **1** |  |  | **1** | **1** | **0** | **0** |  |  | **1** | **0** | **0** | **1** |  |  | **2** | **0** | **0** | **0** |  |  | **0** | **1** | **1** | **0** |  |  |
| **Residence** |  |  |  |  | **3.64** | **0.73** |  |  |  |  | **6.82** | **0.34** |  |  |  |  | **10.32** | **.11** |  |  |  |  | **7.71** | **.3** |  |  |  |  | **5.48** | **.48** |
| In campus | **56** | **46** | **38** | **83** |  |  | **62** | **64** | **51** | **46** |  |  | **67** | **45** | **49** | **62** |  |  | **76** | **28** | **34** | **85** |  |  | **73** | **39** | **33** | **78** |  |  |
| Off campus | **3** | **1** | **0** | **3** |  |  | **1** | **2** | **1** | **3** |  |  | **1** | **3** | **0** | **3** |  |  | **2** | **2** | **2** | **1** |  |  | **3** | **0** | **1** | **3** |  |  |
| Both | **2** | **3** | **2** | **2** |  |  | **5** | **2** | **0** | **2** |  |  | **1** | **1** | **5** | **2** |  |  | **0** | **2** | **2** | **5** |  |  | **4** | **1** | **3** | **1** |  |  |
| **Area of growing** |  |  |  |  | **1.20** | **0.75** |  |  |  |  | **1.96** | **0.58** |  |  |  |  | **4.10** | **.25** |  |  |  |  | **3.8** | **.3** |  |  |  |  | **2.60** | **.46** |
| Rural | **30** | **26** | **17** | **39** |  |  | **30** | **33** | **28** | **21** |  |  | **36** | **25** | **19** | **32** |  |  | **39** | **19** | **17** | **37** |  |  | **33** | **18** | **17** | **44** |  |  |
| Urban | **31** | **24** | **23** | **49** |  |  | **38** | **35** | **24** | **30** |  |  | **33** | **24** | **35** | **35** |  |  | **39** | **13** | **21** | **54** |  |  | **47** | **22** | **20** | **38** |  |  |
| **Level of study year** |  |  |  |  | **13.36** | **0.34** |  |  |  |  | **9.52** | **0.66** |  |  |  |  | **9.06** | **.70** |  |  |  |  | **6.21** | **.9** |  |  |  |  | **15.47** | **.22** |
| First-year | **26** | **15** | **14** | **26** |  |  | **20** | **28** | **17** | **16** |  |  | **21** | **14** | **22** | **24** |  |  | **32** | **8** | **13** | **28** |  |  | **30** | **19** | **8** | **24** |  |  |
| Second-year | **14** | **14** | **11** | **19** |  |  | **18** | **11** | **17** | **12** |  |  | **22** | **11** | **11** | **14** |  |  | **18** | **9** | **9** | **22** |  |  | **18** | **13** | **9** | **18** |  |  |
| Third-year | **16** | **12** | **10** | **29** |  |  | **18** | **19** | **14** | **16** |  |  | **21** | **16** | **12** | **18** |  |  | **19** | **9** | **9** | **30** |  |  | **23** | **3** | **13** | **28** |  |  |
| Fourth-year | **3** | **2** | **3** | **10** |  |  | **5** | **6** | **2** | **5** |  |  | **3** | **4** | **4** | **7** |  |  | **4** | **3** | **4** | **7** |  |  | **4** | **3** | **4** | **7** |  |  |
| Fifth-year | **2** | **7** | **2** | **4** |  |  | **7** | **4** | **2** | **2** |  |  | **2** | **4** | **5** | **4** |  |  | **5** | **3** | **3** | **4** |  |  | **5** | **2** | **3** | **5** |  |  |
| **Family history of mental ill ness** |  |  |  |  | **5.54** | **0.14** |  |  |  |  | **1.65** | **0.65** |  |  |  |  | **3.85** | **.28** |  |  |  |  | **14.38** | **.01** |  |  |  |  | **5.55** | **.14** |
| No | **57** | **42** | **39** | **79** |  |  | **64** | **62** | **46** | **45** |  |  | **65** | **45** | **50** | **57** |  |  | **70** | **32** | **29** | **86** |  |  | **77** | **37** | **32** | **71** |  |  |
| Yes | **4** | **8** | **1** | **9** |  |  | **4** | **6** | **6** | **6** |  |  | **4** | **4** | **4** | **10** |  |  | **8** | **0** | **9** | **5** |  |  | **3** | **3** | **5** | **11** |  |  |
| **Substance use** |  |  |  |  | **3.28** | **0.35** |  |  |  |  | **2.12** | **0.55** |  |  |  |  | **4.72** | **.19** |  |  |  |  | **5.10** | **.2** |  |  |  |  | **0.50** | **.92** |
| No | **56** | **41** | **33** | **78** |  |  | **61** | **58** | **47** | **42** |  |  | **60** | **46** | **43** | **59** |  |  | **67** | **31** | **30** | **80** |  |  | **68** | **35** | **33** | **72** |  |  |
| Yes | **5** | **9** | **7** | **10** |  |  | **7** | **10** | **5** | **9** |  |  | **9** | **3** | **11** | **8** |  |  | **11** | **1** | **8** | **11** |  |  | **12** | **5** | **4** | **10** |  |  |

The association between demographic variables and common barriers to receive professional mental health care
